# Prevalence and antimicrobial susceptibility of extended-spectrum beta lactamases-producing *Escherichia coli* and *Klebsiella pneumoniae* isolated in selected hospitals of Anyigba, Nigeria

**DOI:** 10.4314/ahs.v21i2.4

**Published:** 2021-06

**Authors:** Kehinde C Mofolorunsho, Hannah O Ocheni, Ruth F Aminu, Cornelius A Omatola, Olabisi O Olowonibi

**Affiliations:** 1 Kogi State University, Faculty of Natural Sciences, Department of Microbiology; 2 Tshwane University of Technology, Department of Pharmaceutical Science

**Keywords:** Antibiotic resistance, ESBLs, *Escherichia coli*, *Klebsiella pneumoniae*, Anyigba

## Abstract

**Background:**

*Escherichia coli* and *Klebsiella pneumoniae* are commonly implicated in urinary tract infections accounting for majority of the antimicrobial resistance encountered in hospitals.

**Objectives:**

To determine the prevalence and antimicrobial susceptibility of extended-spectrum beta-lactamases (ESBLs) producing *E. coli* and *K. pneumoniae* among patients in Anyigba, Nigeria.

**Methods:**

This hospital-based cross-sectional study was conducted using urine samples from 200 patients of Grimmard Catholic hospital and Maria Goretti hospital. Urine samples were processed to identify ESBL-producing *E. coli* and *K. pneumoniae* using standard microbiological techniques. Isolates were then tested against antimicrobial agents.

**Results:**

A total of 156 bacterial isolates were recovered consisting 128 of *E. coli* and 28 of *K. pneumoniae*. Extended spectrum beta-lactamases production was observed in 69% of *E. coli* and 31% of *K. pneumoniae*. These pathogens were resistant to 3 or more antibiotics. Of the antimicrobials tested, cefotaxime demonstrated the highest rates of resistance (100%) for both ESBL-producing *E. coli* and *K. pneumoniae*. Fifty-four isolates of ESBL-producing *E. coli* showed a high level of resistance to amoxicillin clavulanic acid (83.3%), ciprofloxacin (83.3%), and ceftazidime (79.6%). ESBL-positive *K. pneumoniae* isolates were highly resistant to ciprofloxacin (75%), and amoxicillin clavulanic acid (83.3%). Cefoxitin (62.5%) and gentamicin (66.7%) showed substantially higher rates of resistance against these isolates while all 24 strains were resistant to imipenem.

**Conclusion:**

This study indicated the prevalence of ESBL-positive Gram-negative pathogens in these study sites and also demonstrated their resistance to a few antibiotics. This highlights the need for new antimicrobials that are potent and improved policy on use of antibiotics.

## Introduction

About 1.5 billion infections due to microorganisms have been reported to occur globally each year resulting in approximately 4.6 million deaths^1^. Several previous studies have highlighted the magnitude of infectious diseases in the human population with reports of an estimated 106 million cases of gonorrhoea, 3.1 million cases of lower respiratory infections and 1.5 million cases of diarrheal diseases globally^2^,^3^. This is worrisome and of major concern especially in hospital settings particularly for critically ill patients and for patients requiring placement of invasive devices or surgical procedures^4^,^5^. The global spread of antimicrobial resistance among bacterial pathogens is a serious threat to human health and a challenge for modern medicine with significant impact on health care cost^6^,^7^. A recent report estimated that 10 million deaths will be attributed to antimicrobial resistance by 2050 and 100 trillion USD of the world's economic outputs will be lost if substantive efforts are not made to contain this threat^8^–^10^. Little wonder the World Health Organization identified antimicrobial resistance as one of the three greatest threats to mankind in the 21^st^ century^11^,^12^. Studies conducted over the years, have identified some Gram-negative pathogens as a major cause of hospital-acquired infections (HAIs) especially in developing countries. These pathogens have accounted for majority of the antimicrobial resistance encountered in hospital settings and has presented serious therapeutic dilemmas for clinicians due to complex resistance profiles resulting in high morbidity and mortality rates as well as prolonged hospital stay^4^,^5^,^13^.

Several resistant Gram-negative bacterial pathogens producing extended-spectrum beta-lactamases (ESBLs) have been increasingly involved in hospital-acquired infections resulting in a dearth of treatment options. Extended-spectrum beta-lactamases (ESBLs) are enzymes encoded on the chromosome or on plasmids, conferring resistance to penicillins, cephalosporins, and monobactams^14^,^15^. The burden of ESBL is currently of global concern to humans, just as is animals and the ecosystem^16^–^19^. Of particular concern is the emergence of ESBL-producing *Klebsiella pneumoniae* in hospital settings^20^ which has considerably increased during the last decade, and ESBL-producing *Escherichia coli*; a leading cause of blood stream and urinary tract infections (UTIs) capable of hydrolysing numerous antibiotics, including third generation cephalosporins^6^. *K. pneumoniae* is the second most common cause of UTIs after *E. coli*, often due to the use of indwelling catheters^21^. Urinary tract infections are one of the most common infections encountered in medical practice affecting a large patient population irrespective of age and gender with a global prevalence estimated to be around 150 million persons per year^22^–^24^.

The World Health Organization's global surveillance report on antibiotic resistance indicated that five out of the six WHO regions had more than 50% resistance to third generation cephalosporins in *E. coli* and *K. pneumoniae* in hospital settings. The report also revealed that *K. pneumoniae* resistant to third generation cephalosporins was associated with elevated deaths in Africa (77%), the Eastern Mediterranean region (50%), South East Asia (81%) and Western Pacific region (72%). It further attributed 45% of deaths in both Africa and South-East Asia to multi-drug resistant (MDR) bacteria. These resistant strains are considered a public health issue^10^,^25^,^26^ and calls for attention.

In Nigeria, indiscriminate use of antibiotics, poor hygiene practices in hospitals settings and the lack of monitoring of antimicrobial resistant microorganisms have been associated with the emergence and uncontrolled spread of ESBLs^27^ resulting in treatment failures, increased morbidity and mortality rates. The objectives of this study were therefore to determine the presence of *E. coli* and *K. pneumoniae* in urine samples collected from hospital patients in Anyigba a community in North Central Nigeria by conventional culture and biochemical analyses. The prevalence and antibiotic susceptibility profile of ESBL-producing clinical isolates were also investigated to ascertain the magnitude of ESBL carriage in order to improve the antibiotic management of hospital-acquired infections in the community.

## Methodology

### Study area

This cross-sectional hospital-based study was conducted in Anyigba, a community in Dekina Local Government of Kogi State, Nigeria. The population of the community which lies between latitude 7o15′-7o29′ north and longitude 7°11′-7°32′ east with an average altitude of 420 m above sea level, is estimated at 130,000^28^.

### Ethical consideration

Ethical approval was obtained from the hospitals' management board on ethics relating to health issues in line with the Declaration of Helsinki on the conduct of biomedical research involving human subjects. All participants gave their consent to enter the study.

### Sample collection

Two hundred samples of urine were collected from both inpatients and out-patients of the Grimmard Catholic Hospital (GCH) and Maria Goretti Hospital (MGH) in Anyigba, North-Central Nigeria between May and October 2018. These frequently utilized private primary healthcare facilities, takes care of the majority of medical cases within the community.

Mid-stream urine samples were collected into sterile universal bottles and immediately transported to the Kogi State University's Microbiology laboratory for analyses. Information on sex and age were obtained from patients' hospital records. All samples were streaked onto MacConkey agar (Oxoid, UK) containing ceftazidime (1mg/L). Incubation was at 37 °C for 24 hours. Culture isolates were then identified based on conventional identification methods including Gram's staining and colony formation. A series of biochemical tests such as catalase, oxidase, urease, indole, citrate utilization, motility, and triple sugar iron test were done.

### Extended-Spectrum Beta Lacatamases Detection

*E. coli* and *K. pneumoniae* were screened and confirmed for extended-spectrum beta lactamases (ESBLs) activity in accordance with Clinical and Laboratory Standards Institute guideline (CLSI)^29^. Initial ESBLs activity was determined by screening cefotaxime (CTX: 30µ g, Oxoid UK), ceftazidime (CAZ: 30µ g, Oxoid, UK), and ceftriaxone (CRO: 30µ g, Oxoid UK) using Mueller Hinton agar (MHA: Oxoid, UK) already inoculated with the isolates.

To improve sensitivity of ESBLs detection, more than one antibiotic disc were used as recommended by CLSI guidelines^29^. Freshly grown colonies were suspended into normal saline and the turbidity of the suspension was adjusted at 0.5 McFarland's standard. The suspension was inoculated onto Mueller Hinton agar (MHA: Oxoid UK) with all three discs place at a gap of 20mm. Plates were then incubated for 18hrs at 37 °C. Isolates with reduced susceptibility to cefotaxime (zone diameter of ≤ 27mm), ceftazidime (zone diameter of ≤ 22mm), and ceftriaxone (zone diameter of ≤ 25mm) around the discs were suspected to be ESBLs producers^29^.

The double discs synergy method was employed for the confirmation of suspected ESBLs producers. This was done by testing the following antibiotic discs; cefotaxime (CTX: 30µ g, Oxoid, UK), ceftazidime (CAZ: 30µ g, Oxoid, UK) and amoxycillin+clavulanic acid (AMC: 30 µ g, Oxoid, UK) on Mueller Hinton agar (MHA: Oxoid, UK). Amoxycillin+clavulanic acid disc was placed in the center of the Mueller Hinton agar plates. Cefotaxime and ceftazidime were placed at a distance of 20mm from the amoxycillin+clavulanic acid disc. Plates were then examined after incubation for 24 hours at 37°C for an expansion of inhibition zone of the oxyimino-β -lactam caused by the synergy of the clavulanate in the amoxycillin+clavulanic acid disc which was interpreted as ESBLs positive.

### Antibiotic Susceptibility Test

Susceptibility testing of isolates to 7 antibiotics was performed using disc diffusion method. Isolates were enriched in peptone water for 24 hours after which 0.1ml was streaked onto Mueller Hinton agar (Oxoid, UK). The following antibiotics were used; cefoxitin (FOX: 30µ g, Oxoid, UK), cefotaxime (CTX: 30µ g, Oxoid, UK), ceftazidime (CAZ: 30µ g, UK, Oxoid), gentamicin (GEN: 10µ g, Oxoid, UK), ciprofloxacin (CIP: 5µ g, Oxoid, UK), amoxycillin+clavulanic acid (AMC: 30µ g, Oxoid, UK) and imipenem (IPM: 10µ g, Oxoid, UK). Results were interpreted as resistant or susceptible based on the interpretative standard according to the clinical and laboratory standards institute (CLSI) manual29.

## Results

### Demographic characteristics of patients

Table 1 shows the age and sex distribution of participants. The age range of patients who participated in the study was between 17 to 72 years. Study population was predominantly females (66.0%) with a male to female ratio of 1:1.9. During the study period, 200 urine samples were analysed. Forty-four samples were excluded because the causative pathogens of interest could not be identified. Consequently, 156 (78.0%) cases were included in this study. The common most predominant causative pathogen was *E. coli* which accounted for 128 (82.1%) cases. More isolates (57.7%) were recovered from females with the least proportion of bacteria (*K. pneumoniae*) isolated in the male population (Table 2).

**Table 1:** Age and sex distribution of participants

Age group (Years)	Male (%)	Female (%)	Total (%)
17 – 21	8	28	36
22 – 26	0	36	36
27 – 31	16	12	28
32 – 36	12	24	36
37 – 41	8	20	28
42 – 46	8	4	12
47 – 51	12	4	16
>51	4	4	8
**Total**	68 (34.0)	132 (66.0)	200 (100)

**Table 2:** Distribution of isolates among gender and study sites

Characteristic	*E. coli (%)*	*K. pneumoniae (%)*	Total (%)
**Sex**			
Male	56 (43.8)	10 (35.7)	66 (42.3)
Female	72 (56.2)	18 (64.3)	90 (57.7)
**Study site**			
MGH	90 (70.3%)	20 (71.4%)	110 (70.5%)
GCH	38 (29.7%)	08 (28.6%)	46 (29.5%)

### Frequency of Isolates

The frequency of isolates in relation to study sites is shown in table 3. A total number of 110 (70.5%) isolates of both *E. coli* and *K. pneumoniae* were recovered from MGH while 46 (29.5%) isolates (*E. coli* and *K. pneumoniae*) were obtained from GCH. Higher percentages of *E. coli* (70.3%) and *K. pneumoniae* (71.4%) isolates recorded, were also from MGH.

**Table 3:** Antimicrobial susceptibility profile of ESBL isolates

		ESBL-producing bacteria (n = 78)	
		*E. coli (%)*	*K. pneumoniae (%)*
**Cefotaxime**	Resistance Intermediate Sensitive	54 (100) - -	24 (100) - -
**Cefoxitin**	Resistance Intermediate Sensitive	12 (22.2) 04 (7.4) 38 (70.4)	15 (62.5) 05 (20.8) 04 (16.7)
**Ceftazidime**	Resistance Intermediate Sensitive	43 (79.6) 02 (3.7) 09 (16.7)	10 (41.7) 03 (12.5) 11 (45.8)
**Imipenem**	Resistance Intermediate Sensitive	12 (22.2) 39 (72.2) 03 (5.6)	24 (100) - -
**Amoxycillin+clavulanic**	Resistance Intermediate Sensitive	45 (83.3) 09 (16.7) -	20 (83.3) 01 (4.2) 03 (12.5)
**Ciprofloxacin**	Resistance Intermediate Sensitive	45 (83.3) - 09 (16.7)	18 (75.0) 04 (16.7) 02 (8.3)
**Gentamicin**	Resistance Intermediate Sensitive	27 (50) 15 (27.8) 12 (22.2)	16 (66.7) 04 (16.7) 04 (16.7)

### Isolation of ESBL-producing bacteria

Expression of ESBLs was phenotypically detected by double discs synergy test methods. The total number of ESBL-producing isolates was 78 (Table 3). Out of these ESBL-producing isolates, *E. coli* accounted for 69% (54/78) whereas *K. pneumoniae* accounted for 31% (24/78).

### Antimicrobial Resistance of ESBL Isolates

Majority of all ESBL-producing isolates displayed phenotypic resistance to three or more drugs. *Escherichia coli* isolates were found to be highly resistant to both beta lactam (cefotaxime, ceftazidime and amoxicillin clavulanic acid) and non-beta lactam (ciprofloxacin) antibiotics. Resistance to ciprofloxacin and gentamicin accounted for 83.3% and 50% respectively. All isolates of *K. pneumoniae* exhibited a 100% resistance to cefotaxime and imipenem. Isolates were also resistant to ciprofloxacin (75%), cefoxitin (62.5%), gentamicin (66.7) and amoxicillin clavulanic acid (83.3%). Results on the antimicrobial susceptibility of these ESBL-producing isolates are summarized in table 3.

## Discussion

The emergence and rapid spread of multi-drug resistant pathogens are of great concern worldwide; among them, ESBL-producing *Enterobacteriaceae* has been a major concern. During the past decades, ESBL-producing Gram-negative bacteria especially *E. coli* and *K. pneumoniae* have emerged as serious pathogens both in hospital and community acquired infections worldwide^30^. Developing countries are far behind in the fight against antimicrobial resistance with considerable efforts needed to reduce morbidity and mortality due to infection caused by these multi-drug resistant pathogens^31^–^33^. Therefore, bacterial infection and antibiotic resistance surveillance are essential for effective management of infections.

In this study, the number of male patients to female patients ratio was 1:1.9. This concurred with findings from Nepal and Nigeria, that reported similar ration among patients suspected to have urinary tract infections^30^,^34^. This increase in the number of female participants may be due to involuntary recruitment bias. This study, similar to other studies,^34^ further showed that females were most affected among positive cases with higher isolation rates. Several predisposing factors such as increase in age, frequency of sexual activities and high parity have been attributed to the high infection rates among female patients^35^–^38^. Also, the short length of the urethra and it proximity to the anus, makes colonization with colonic Gram-negative bacteria possible^39^.

Findings in this study showed a high prevalence of infections among patients attending MGH (70.5%) compared to patients in GCH. The high prevalence reported in this study site can be attributed to higher intake of patients due to its location within a university community thus receiving higher numbers of patients.

In our study, *E. coli* was the predominant pathogen isolated with an isolation rate of 82.1%. *K. pneumoniae* accounted for 17.9% of infection. This finding is consistent with previous studies which indicated that *E. coli* and *K. pneumoniae* were among Gram-negative pathogens associated with about 90% of both community and hospital acquired UTIs^40^,^41^. These bacterial pathogens have been associated with attributable mortality due to their high antibiotic resistance and thus categorised by the World Health Organization (WHO) as critical Gram-negative pathogens under surveillance.^10^ Other studies have reported *E. coli* as the most prevalent clinically important pathogen implicated in UTIs followed by *K. pneumoniae*^42^–^45^.

The true prevalence of ESBL is not well-known in Africa because of the paucity of studies in human and animal health. Nonetheless, studies have found that ESBL-producing bacteria are common and vary between countries of the continent^46^,^47^.

In our study, we identified 50% (78/156) of all isolates as ESBL-producers. This high rates observed is in contrast to previous studies^48^,^49^ where rates were reported to be as low as 2% in the Netherlands, 2.6% in Germany and 16% in Nigeria. However, studies by^50^,^51^ reported high rates of ESBL production. This observed rate may be attributed to the practices of self-medication and the less controlled use of antibiotics which are available over-the-counter in this region. Additionally, regulations promoting rational use of antibiotics are minimal or non-existent^26^,^52^. Sixty-nine per cent (54/78) of *E. coli* and 31% (24/78) of *K. pneumoniae* were identified as ESBL-producers and found to show resistance to three or more antibiotics.

Our study clearly revealed high resistance rates of ESBL- producing *E. coli* to ceftazidime (79.6%), cefotaxime (100%), amoxicillin clavulanic acid (83.3%) and ciprofloxacin (83.3%). This results are in agreement with studies conducted in Thailand where high resistance to ciprofloxacin, ceftazidime and cefotaxime were reported^53^,^54^. The high resistance observed in this study calls for serious concerns considering the fact that antibiotic use is less controlled in this sub-region. This gives room for self-medication and abuse due to easy availability of these antibiotics^55^.

ESBL-producing *K. pneumoniae* isolates were found to be resistant to ciprofloxacin, cefoxitin, gentamicin, imipenem and amoxicillin clavulanic acid. All 24 isolates were resistant to cefotaxime. These findings are consonant with other research^56^. Carbapenems are regarded as the drugs of choice for treatment of infections caused by ESBL-producers. However, reports have indicated that carbapenemase producing Enterobacteriaceae isolates seem to be increasing in number in the last few years^57^,^58^,^59^. In this study, the tested ESBL-producing *K. pneumoniae* isolates showed high resistance rate for imipenem (100%). This is in close conformity with the findings in the study conducted by Ferreira et al.^59^ from Brazil who reported 100% of *K. pneumoniae* strains were carbapenemase producers. A similar study by Nagaraj et al.^60^ also showed a resistance of 75% to carbapenems. This study highlights a worrying prevalence of ESBL-producing Gram-negative bacteria associated with urinary tract infections. Immediate action is therefore needed to prevent these resistant bacteria from spreading in both healthcare and community settings. Sustainable efforts at developing new antibiotics and vaccines should be encouraged to advance the containment of this threat.^26^ High antibiotic usage should also be reduced. This can be achieved through stewardship and guidance on appropriate use. Furthermore, routine surveillance of antimicrobial resistant isolates should be incorporated.
